# Drug combination sensitivity scoring facilitates the discovery of synergistic and efficacious drug combinations in cancer

**DOI:** 10.1371/journal.pcbi.1006752

**Published:** 2019-05-20

**Authors:** Alina Malyutina, Muntasir Mamun Majumder, Wenyu Wang, Alberto Pessia, Caroline A. Heckman, Jing Tang

**Affiliations:** 1 Institute for Molecular Medicine Finland (FIMM), Helsinki Institute of Life Science, University of Helsinki, Helsinki, Finland; 2 Research Program in Systems Oncology, Faculty of Medicine, University of Helsinki, Helsinki, Finland; University at Buffalo - The State University of New York, UNITED STATES

## Abstract

High-throughput drug screening has facilitated the discovery of drug combinations in cancer. Many existing studies adopted a full matrix design, aiming for the characterization of drug pair effects for cancer cells. However, the full matrix design may be suboptimal as it requires a drug pair to be combined at multiple concentrations in a full factorial manner. Furthermore, many of the computational tools assess only the synergy but not the sensitivity of drug combinations, which might lead to false positive discoveries. We proposed a novel cross design to enable a more cost-effective and simultaneous testing of drug combination sensitivity and synergy. We developed a drug combination sensitivity score (CSS) to determine the sensitivity of a drug pair, and showed that the CSS is highly reproducible between the replicates and thus supported its usage as a robust metric. We further showed that CSS can be predicted using machine learning approaches which determined the top pharmaco-features to cluster cancer cell lines based on their drug combination sensitivity profiles. To assess the degree of drug interactions using the cross design, we developed an S synergy score based on the difference between the drug combination and the single drug dose-response curves. We showed that the S score is able to detect true synergistic and antagonistic drug combinations at an accuracy level comparable to that using the full matrix design. Taken together, we showed that the cross design coupled with the CSS sensitivity and S synergy scoring methods may provide a robust and accurate characterization of both drug combination sensitivity and synergy levels, with minimal experimental materials required. Our experimental-computational approach could be utilized as an efficient pipeline for improving the discovery rate in high-throughput drug combination screening, particularly for primary patient samples which are difficult to obtain.

## Author summary

Cancer is one of the main causes of death worldwide. Although new treatment strategies have been achieved, they still have limited efficacy as cancer cells can easily develop drug resistance. To achieve more sustainable therapies to treat cancer, we need multi-targeted drug combinations that can inhibit cancer cells more effectively and synergistically. However, the increasing number of possible drug combinations makes a full matrix design unfeasible, even with automated drug screening instruments. Therefore, we proposed a novel cross design to access drug combinations more efficiently. We further developed a drug combination sensitivity score (CSS) that is tailored for the cross design to quantify the efficacy of a drug combination. Using public datasets, we showed that the CSS is a robust metric and highly predictive with an accuracy comparable to the experimental replicates. We also developed a CSS-based synergy score to assess the degree of drug interaction and showed its capability to correctly identify synergistic and antagonistic drug combinations. Taken together, we showed that the cross design and its scoring methods allow a more systematic and cost-effective evaluation of drug combinations. The proposed experimental and computational techniques are expected to be widely applicable in the field of drug combination discovery.

This is a *PLoS Computational Biology* Methods paper.

## Introduction

Despite great advances in the understanding of cancer, there remains a major challenge to develop more effective anti-cancer treatments. Next generation sequencing has revealed the intrinsic heterogeneity in cancer genomes, which partly explains why patients respond differently to the same therapy [1]. To reach durable clinical responses, cancer patients who relapse and become refractory to standard chemotherapy need novel multi-targeted drug combinations which can effectively overcome the emergence of drug resistance [2–4]. Ideally, a potential drug combination should achieve therapeutic efficacy at reduced dosages, and therefore minimize the toxicity and other side effects associated with high doses of single drugs [5–6]. Therefore, two important properties for a drug combination must be evaluated: sensitivity and synergy. Sensitivity of a drug combination is defined as the level of treatment response, usually measured in the unit of percentage inhibition of cell viability or growth. In contrast, synergy of a drug combination is referred to the degree of drug interactions that contributes to the drug combination sensitivity independent of the single drug effects [7].

In order to identify sensitive and synergistic drug combinations, high-throughput drug screening has been applied on a large variety of cancer cell lines and more recently on patient-derived cancer samples [8–9]. Many high-throughput drug combination screens test pairs of drugs at a dose matrix, for which the cell viability or growth inhibition effects are measured [10–11]. The dose-response matrix results from a full factorial design that involves multiple dose combinations of a drug pair, and thus demands a relatively large amount of cancer cells. For patient-derived cancer samples, which are challenging to obtain and restricted in volume, the full matrix design may be infeasible to test even with a minimal number of drug combinations. Furthermore, cancer samples of different genetic profiles are known to respond differently to the same treatment [12]. With the limited amount of drug combination data points, it becomes a daunting task for any machine learning approach to navigate the combinatorial space to pinpoint the most promising drug combinations that are selectively effective for individual cancer samples [13].

Furthermore, many existing computational tools for drug combination analysis focus on the degree of interaction, i.e. drug synergy, but not the sensitivity of drug combinations. For example, Combenefit [14] and SynergyFinder [15] have been developed to provide multiple reference models to score drug synergy, such that drug combinations that produce higher growth inhibition effects compared to the single drugs will be prioritized. There have been multiple methods to score drug synergy [16], while the choice of the best synergy scoring methods is under debate [7,17]. On the other hand, as synergy is a measure of drug interaction while sensitivity is a measure of drug combination efficacy, these two metrics are expected to capture distinct properties of a drug combination. Therefore, neglecting the drug combination sensitivity may lead to a biased prioritization of drug combinations that are unable to kill cancer cells despite strong synergy [18]. However, unlike the sensitivity of single drugs which can be directly derived from monotherapy dose-response curves [19], the sensitivity of a drug combination remains largely undefined, as the same sensitivity can be achieved using different dose combinations. Furthermore, there is a lack of scoring approaches to fully capture the synergy and sensitivity simultaneously, which should be ideally interpretable using the same scale, e.g. percentage inhibitions [16].

To overcome these challenges, we proposed a cost-effective experimental and computational procedure to facilitate the prioritization of drug combination synergy and sensitivity. We proposed a novel experimental design to allow either drug to span over multiple doses while the concentration of the other drug is fixed at its IC_50_ concentration. The resulting drug combination dose-response curves were utilized to determine a drug combination sensitivity score (CSS). Using a large-scale drug combination study, referred to as the O’Neil data [20], we showed that the CSS is highly reproducible, suggesting its robustness to be utilized as a metric for characterizing drug combination responses. Furthermore, we found that the CSS can be predicted at high accuracy using chemical and pharmacological features of the drug combinations. To assess the degree of synergy from the cross design, we developed an S synergy score based on the difference between the observed CSS score and the baseline effect predicted by a reference model. As there is no consensus on the reference model, we evaluated multiple variants of it and found that the S score can detect the true synergistic and antagonistic drug combinations with high accuracy, irrespective of which the reference model is used. Compared to the full matrix design, the cross design requires minimal amount of experimental materials, while it still maintains a robust and accurate characterization of both drug combination sensitivity and synergy levels. We foresee that such a cross experimental design and its CSS and S scoring methods should allow a scale-up of drug combination testing especially for patient-derived cancer cells. The R scripts for calculating and predicting CSS are available at https://github.com/amalyutina/CSS.

## Materials and methods

### The cross drug combination design

**Fig 1:**
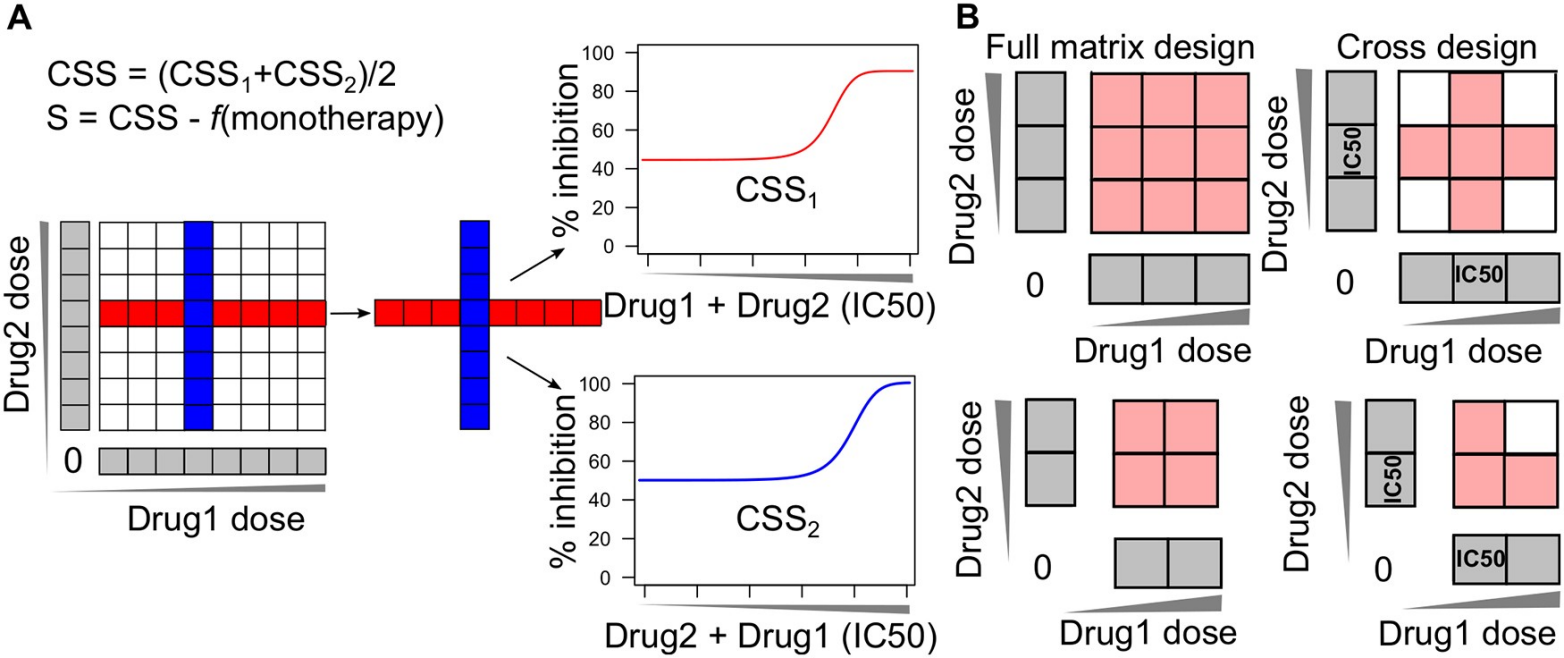
Drug combination cross design. (A) The cross design to determine the drug combination sensitivity score. Compared to the full matrix design (left panel), only a single row and a single column from the matrix that correspond to the IC_50_ concentrations of the two drugs are utilized for the calculation of CSS (middle panel). One drug is utilized as the background drug fixed at its IC_50_ concentration while the other drug becomes the foreground drug with multiple doses being titrated. The resulting two dose-response curves will be summarized as the drug combination sensitivity score (CSS), from which the S synergy score can be determined as the deviation from a reference model which predicts the expected percentage inhibition effect from monotherapy dose responses. (B) Comparing the cross design with the full matrix design in terms of data size. Less materials are needed for the cross design when the size of the full matrix is two or above.

We proposed a cross design to test the synergy and sensitivity of a drug pair by first introducing the concepts of background drug and foreground drug: background drug is the drug fixed at its IC_50_ concentration while foreground drug is added into the background drug with multiple concentrations. We allow either drug in the pair to be the background drug, so that two vectors of dose combinations will be intersected at the IC_50_ concentrations (Fig 1A). The dose-response curves for these two vectors are usually measured in a unit of inhibition percentages by cell viability or toxicity assays. Note that the cross design requires specifically the combinations at the IC_50_ concentrations, which need to be determined based on the monotherapy dose response curves. As shown in Fig 1B, as long as a minimal of two concentrations are tested for a drug combination, the cross design will require less experimental materials than a full matrix design. For a combination screen that tests one concentration only, both the cross design and the full matrix design converge to a point estimate of drug combination effects at IC_50_. Therefore, we considered that the cross design is in general more cost-effective compared to the full matrix design.

### Determination of the CSS drug combination sensitivity scores

With the drug combination dose-response curves determined in the cross design, the CSS summarizes the area under the curve similar to the scoring approaches [21–22]. Namely, a four-parameter log-logistic function is used to fit the dose-response curve for a concentration *x* of the foreground drug according to:

$$y=y_{min}\;+\frac{y_{max}-y_{min}}{1+10^{\lambda ({{log}_{10}IC}_{50}-{log}_{10}x)}},$$ (1)

where *y*_*min*_ and *y*_*max*_ are the minimal and maximal percentage inhibition (the bottom and top asymptotes of the curve, 0 ≤ *y*, *y*_*min*_, *y*_*max*_ ≤ 1); IC_50_ is the concentration of the foreground drug with which the drug combination reaches 50% of *y*_*max*_*—y*_*min*_ inhibition of the cell growth; λ is the slope of the dose-response curve.

The dose-response curve (1) is transformed by substituting x with *x'* = log_10_(*x*) as:

$$y=y_{min}\;+\frac{y_{max}-y_{min}}{1+10^{\lambda ({{log}_{10}IC}_{50}-x^{'})}}$$ (2)

The area under the log_10_-scaled dose-response curve (AUC) is determined according to

$$AUC=\int_{c_{1}}^{c_{2}}y_{min}+\frac{y_{max}-y_{min}}{1+10^{\lambda (m-x^{'})}}dx^{'}=y_{min}(c_{2}-c_{1})+(y_{max}-y_{min})\frac{1}{\lambda }{log}_{10}(\frac{1+10^{\lambda (c_{2}-m)}}{1+10^{\lambda (c_{1}-m)}}),$$ (3)

where [*c*_*1*_, *c*_*2*_] is the log_10_ concentration range for the foreground drug tested in the experiment, and *m = log*_*10*_*(IC*_*50*_*)*.

The AUC is further normalized as the proportion of its maximal possible inhibition (i.e. 100% inhibition) according to:

$${AUC}^{'}=\frac{AUC–{inh}_{min}(c_{2}-c_{1})}{\left(1-{inh}_{min})(c_{2}-c_{1}\right)},$$ (4)

where *inh*_*min*_ is the minimum inhibition level that is considered as the drug effect (by default it is fixed at 10%, assuming that the inhibition below 10% is experimental noise, S1 Fig).

The CSS for the foreground drug is defined as a percentage which varies between 0 and 100:

$$CSS=100{AUC}^{'}$$ (5)

As there are two drug combination dose-response curves depending on which drug is fixed as the background drug, we refer to the results of Eq (5) for either scenario as CSS_1_ and CSS_2_. The two variants of CSS are expected to reflect similarly the summarized % inhibition for a given drug combination. We considered them as two samples that are generated from the same random variable, and estimated the CSS as an average of CSS_1_ and CSS_2_, i.e.

$$CSS=\frac{\left({CSS}_{1}+{CSS}_{2}\right)}{2}$$ (6)

### The O’Neil drug combination data

Dose-response was measured as percentage of cell viability and retrieved from the supplementary material of O’Neil et al. [20], which includes 22,737 drug combinations that involve 38 unique drugs in 39 cancer cell lines, representing 7 tissue types. At the first stage, single-drug screening was done using 8 concentrations to determine the IC_50_ concentration for each drug with six replicates. At the second stage, a 4 by 4 dose matrix was utilized to cover the span of IC_50_ concentrations for a drug pair with four replicates. To utilize the cross design, we picked up only the row and the column corresponding to the concentrations closest to the IC_50_ of the single drugs. These two vectors thus allowed the fitting of drug combination dose-response curves with which the CSS can be calculated. The cell viability percentage was first transformed to inhibition percentage according to:

%inhibition=100-%viability (7)

In our analysis, the CSS for a drug combination was determined based on the average % inhibition of the four replicates. The robustness of the CSS scoring was assessed using the Pearson correlation across the four replicates. All the correlation analyses utilized Pearson correlations.

### Predicting the CSS using machine learning approaches

With the CSS being determined for each drug combination, we sought to evaluate the prediction accuracy of multiple machine learning methods. We considered a drug combination as a combination of their targets and chemical fingerprints. We collected the known targets that have been experimentally validated for the 38 drugs from Drugbank [23] and ChEMBL [24]. Furthermore, we also utilized the Similarity Ensemble Approach (SEA) to predict additional secondary targets based on the chemical structures of the drugs [25]. The targets that were predicted with Z-score higher than 20, Tanimoto coefficient higher than 0.4 and P-value smaller than 0.01 were included, following the previously reported filtering strategy [25]. The MACCS fingerprints of the drugs were determined using the SMILES strings with the R package *rcdk* [26]. The resulting feature set for a single drug included 398 validated and predicted targets and 166 MACCS fingerprints (S1 and S2 Tables). The feature vector for a drug combination was determined as the bitwise OR operation over the features of its single drugs.

We compared three state-of-the-art machine learning methods for the CSS prediction: Elastic Net [27], Random Forests [28] and Support Vector Machines [29]. Elastic Net is a regularization and feature selection method that combines both ridge and lasso regression by including the L_1_ and L_2_ penalty terms, which are regulated by hyper parameters α and λ. The α parameter controls the penalty term in the elastic net by giving more power either to the lasso regression (when α is closer to 1) or to the ridge regression (when α is closer to 0). In our studies, α was selected from the interval [0.1, 1], which determines the level of compromise between the lasso and ridge regressions. The λ parameter regulates the level of shrinkage and was chosen to minimize the difference between predicted and actual CSS scores. Random Forests is an ensemble learning method that constructs multiple decision trees. In our studies, we set the number of randomly selected predictors that is used at each split of the decision tree equal to the rounded down square root of the number of variables. We utilized Support Vector Machines with Radial Basis Function Kernel, which can be used not only for classification but for regression problems. The tuning parameters are the cost parameter *C* that sets the penalty for prediction error of a training point and a smoothing parameter σ, based on the loss function in cross-validation.

We focused on the model performance for predicting new drug combinations within the same cell line, as the set of drug combinations in the training data did not overlap with that in the test data. For each of the 39 cell lines, the number of drug combinations ranges from 290 to 688, with an average of 338. We randomly sampled 70% of the drug combinations to train multiple machine learning models using 10-fold cross-validation, which splits the training data randomly into 10 equally folds, 9 of which were used to fit the model and the remaining one was used to evaluate the prediction accuracy. The model with the lowest RMSE out of the 10-fold cross validation was then used for predicting the CSS values for the remaining 30% of the novel drug combinations as the testing data. As the sampling was done randomly for each cell line, the training and the testing data were therefore balanced. Four metrics including coefficient of determination (R2), root mean square error (RMSE), mean absolute error (MAE) and Pearson correlation (COR) were utilized for evaluating the prediction performance on the testing data. The whole procedure above was repeated 20 times for each cell line on the predefined seeds, and the final model performance was obtained as the mean values of these iterations. To benchmark the performance of the machine learning methods, we utilized one randomly selected technical replicate as the best possible prediction to obtain the upper limit of the performance. All the methods were implemented and evaluated using the R package *caret* [30].

### Determination of the S drug synergy scores

The advantage of CSS is that it allows a direct comparison of the sensitivity between a drug combination and its single drugs, and hence facilitates the quantification of drug synergy. The degree of synergy is often calculated as the deviation of the observed drug combination effect from the reference, which is defined as the expectation effect if the drugs are not interacting. However, how much the expected effect should be is a matter of mathematical modelling with certain assumptions. As the choice of the ‘best’ synergy model is rather heuristic, we proposed three variants of CSS-based synergy scores (termed as S scores) by assuming the reference model as the sum, the maximal and the mean of the AUCs for the monotherapy drug responses:

$$S_{sum}=CSS-sum({AUC}_{1},{AUC}_{2}),$$ (8)

$$S_{max}=CSS-max({AUC}_{1},{AUC}_{2}),$$ (9)

$$S_{mean}=CSS-mean({AUC}_{1},{AUC}_{2}),$$ (10)

The AUC for a monotherapy drug response was defined according to [22]:

$$AUC=\frac{a(x-c+\frac{{log}_{10}(1+10^{b(c-x)})}{b})}{{log}_{10}a},$$ (11)

where [a, b, c] were the parameters to fit a logistic function on the single drug response at concentration x:

$$y=\frac{a}{1+10^{b(c-x)}}$$ (12)

To evaluate the prediction accuracy of the S synergy scores, we defined a set of true synergistic and antagonistic drug combinations as the gold standard, which were determined using the full dose-response matrix data including the combination and monotherapy responses. We utilized the R package *synergyfinder* [31] to calculate multiple versions of synergy scores including the HSA (Highest Single Agency [32]), the Bliss [33], the Loewe [34] and the ZIP synergy scores [35]. The principles of these four models are summarized below:

Consider that drug 1 at concentration *x*_*1*_ and drug 2 at concentration *x*_*2*_ were combined to produce the inhibition effect of *y*_*c*_, while their respective single drug effects were *y*_*1*_(*x*_*1*_) and *y*_*2*_(*x*_*2*_). The synergy score was calculated as the difference between *y*_*c*_ and the expected effect *y*_*e*_ if there is no synergy. Each synergy scoring took a different model for *y*_*e*_:

1. HSA: *y*_*e*_ is the maximal single drug effect, defining
  $$S_{HSA}=y_{c}-max\;(y_{1}(x_{1}),\;y_{2}(x_{2}))$$ (13)
2. Bliss: *y*_*e*_ is the expected effect of two drugs acting independently, defining
  $$S_{Bliss}=y_{c}-(y_{1}(x_{1})+\;y_{2}(x_{2})-\;y_{1}(x_{1})y_{2}(x_{2}))$$ (14)
3. Loewe: *y*_*e*_ is the expected effect of a drug combined with itself, defining
  $$S_{Loewe}=y_{c}-y_{1}(x_{1}+\;x_{2})=y_{c}-y_{2}(x_{1}+\;x_{2})$$ (15)
4. ZIP: *y*_*e*_ is the expected effect of two drugs that do not potentiate each other, defining
  $$S_{ZIP}=y_{c}^{'}-(y_{1}^{'}(x_{1})+\;y_{2}^{'}(x_{2})-\;y_{1}^{'}(x_{1})y_{2}^{'}(x_{2})),$$ (16)
  where $y_{c}^{'},y_{1}^{'}(x_{1})$ and $y_{2}^{'}(x_{2})$ are the fitted values based on the full-dose response matrix for the combination and monotherapy drugs, respectively.

For each of the four models, the synergy scores were determined first for a given dose combination and then were averaged over the full dose-response matrix. With the four synergy scores determined for each drug combination, the true synergistic and antagonistic drug combinations are those with all four synergy scores consistently higher than 5 and lower than 5, respectively. The aim was then to use the S synergy score which was determined by the cross design data to predict the ground truth determined by the full matrix design. The areas under the ROC curve and the precision-recall curve were used for evaluating how well the S synergy scores can predict the consensus drug combinations determined using the full dose-response matrix data.

## Results

### CSS values are highly reproducible and robust

**Fig 2:**
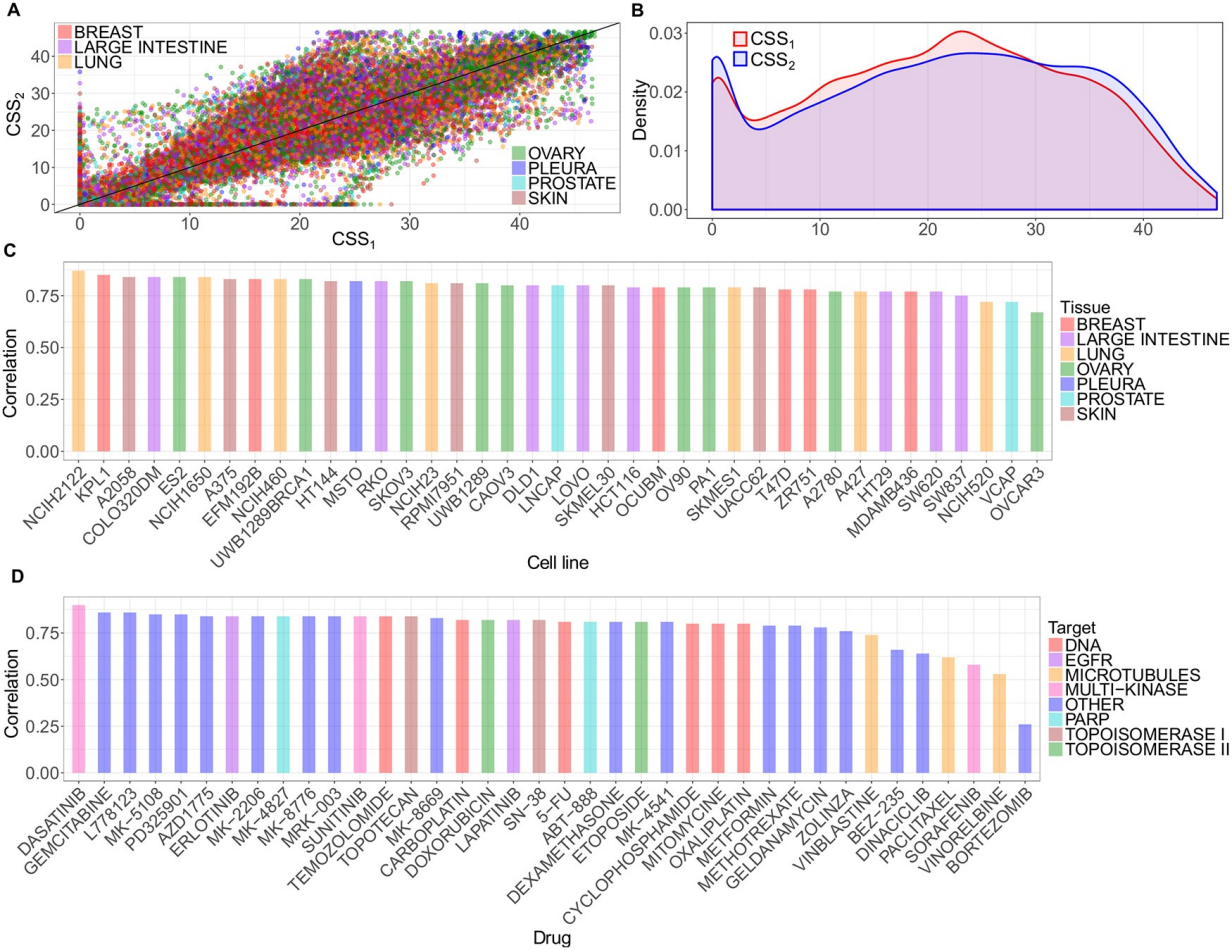
Robustness and replicability of CSS. (A) The Pearson correlation of CSS_1_ and CSS_2_ over all the drug combinations colored according to tissue type; (B) Density plot of the CSS_1_ and CSS_2_ distributions; (C) The Pearson correlation per cell line colored according to tissue type; and (D) The Pearson correlation per drug colored according to drug target class.

We applied the CSS scoring on the O’Neil drug combination data, which consists of 22,737 drug combinations for 39 cancer cells [20]. We found that the CSS_1_ and CSS_2_ values calculated using either drug fixed at its IC_50_ concentration were highly correlated (Pearson correlation = 0.82, p-value = 2×10^−16^; Fig 2A). Both CSS_1_ and CSS_2_ values ranged from 0 to 50, with a marginal absolute difference of 5.62 (Fig 2B). As a CSS score can be directly interpreted as a normalized average % inhibition of the drug combination response (Eqs 1–6), such a result implies that about 5% inhibition difference is expected between CSS_1_ and CSS_2_. The high level of consistency holds true for all the 39 cancer cell lines and the majority of the 38 unique drugs, suggesting the robustness of the CSS scoring method (Fig 2C and 2D, S2 Fig). To evaluate the robustness of the CSS values further, we permuted the O’Neil data randomly and recalculated the correlations between CSS_1_ and CSS_2_. The correlations deteriorated quickly to near zero (Pearson correlation = 0.075, S3 Fig), suggesting that the high correlation can be attributed to the robustness of CSS on the actual drug combination data. We also found high correlation between the CSS value and those derived from individual replicates (minimal Pearson correlation = 0.97, S3 Table). In order to check whether the CSS values are within the range of the CSS replicates for each drug combination, we calculated the minimal and maximal values over the CSS replicates for each drug combination and plotted them together with CSS values over the standard deviation of the CSS replicates. For a better visualization, we applied a generalized additive model to smoothen the CSS lines and obtain 95% pointwise confidence interval around the mean (S4 Fig). Only 4% of the drug combinations have the CSS values being out of the CSS replicate-based limits, however this can be explained by the higher variance over the replicates.

Notably, we found that drug combinations that involved bortezomib showed much lower correlation (0.26) between the CSS_1_ and CSS_2_ values compared to other drug combinations. Since the O’Neil data contains the replicates for single drug screening, we analyzed the coefficient of variation (CV) of the cell viability readout for each drug in the replicates. As expected, we found that bortezomib has the highest CV (0.26), suggesting a relative low quality of the drug combination sensitivity data involving this drug (S5 Fig). When filtering out the drug combinations with a decreasing threshold of difference between CSS_1_ and CSS_2_, the remaining drug combinations showed an increasing CSS_1_ and CSS_2_ correlation (S6 Fig). We found that the threshold of 10 is close to the middle point that reached the correlation of 0.91 (i.e. the average of 1 and 0.82). We therefore applied an empirical threshold of 10 to filter out the drug combinations that were in poor quality, as CSS_1_ and CSS_2_ in these combinations showed bigger than 10% inhibition difference. In total, there were 18,905 drug combinations after the filtering, constituting the 83.1% of the original data. As expected, the correlation between CSS_1_ and CSS_2_ was further improved (Pearson correlation = 0.93, p-value = 2×10^−16^). Furthermore, the mean absolute difference between CSS_1_ and CSS_2_ was 3.83, which was comparable to the variability determined from the technical replicates of CSS_1_ and CSS_2_ (2.92 and 3.06 respectively), suggesting that the difference between CSS_1_ and CSS_2_ is similar to what is expected when repeating the experiment. Taken together, CSS_1_ and CSS_2_ values are highly consistent and therefore supported their averaging as a summary for the drug combination sensitivity score.

### CSS can be predicted using machine learning approaches

Given that the CSS is highly reproducible as a summary of the overall sensitivity of a drug combination, we explored whether CSS can be predicted using pharmacological and chemical information of the drugs. We considered a drug combination as a combination of its drugs’ target profiles as well as their chemical fingerprints, with which the machine learning approaches illustrated in the previous section can be optimized by exploring the feature space using the training data. We examined three major machine learning methods for predictions: Elastic Net, Random Forests and Support Vector Machines.

We found that all of these machine learning approaches worked reasonably well, where Elastic Net consistently achieved the best performance, with a mean MAE of 4.01 which is comparable to that (2.07) of a technical replicate (Table 1). Note that in our cross-validation setting the drug combinations in the test data were not present in the training data, however, the machine learning methods were still able to predict the CSS values for new drug combinations by exploring the feature similarity in the drug targets and chemical fingerprints. The prediction performance thus validated our hypothesis that a drug combination can be considered as a combination of their drug target profiles and chemical-structural properties, with which the CSS score can be predicted with high confidence using state-of-the-art machine learning approaches.

**Table 1:** The prediction performance for Elastic Net, Random Forests and Support Vector Machines, as compared to the upper limit when randomly selecting one technical replicate as the prediction. RMSE: root mean square error, R2: coefficient of determination, COR: Pearson correlation, MAE: mean absolute error.

*Method*	*RMSE*	*R2*	*COR*	*MAE*
*Elastic Net*	5.20±1.11	0.80±0.06	0.90±0.03	4.01±0.86
*Random Forests*	6.30±1.18	0.71±0.07	0.85±0.04	4.75±0.9
*Support Vector Machines*	7.47±1.32	0.57±0.08	0.80±0.04	5.80±1.07
*Technical replicate*	2.87±0.59	0.93±0.04	0.97±0.02	2.07±0.45

All the values are mean+/-standard deviation.

Since both the drug target profiles and chemical fingerprints were considered as the drug combination features, we next evaluated their prediction performances separately using the Elastic Net method. For drug-target profiles we collected known targets that were experimentally validated as well as the additional secondary targets that were predicted with high confidence using the SEA method. For chemical fingerprints we used the MACCS fingerprint which contains 166 structural features [36]. As expected, when combining all the features the model achieved the best performance (Table 2). We found that in general drug target profiles were predictive of CSS, especially when including the experimentally validated targets. The predicted targets using the SEA method did not improve the prediction accuracy significantly, indicating that even though secondary drug target interactions may occur, most likely they have minor functional impact that may not lead to changes in cancer cell viability and thus does not contribute to the prediction of CSS. On the other hand, we found that chemical fingerprints were less predictive of CSS compared to the drug-target profiles, suggesting that the use of MACCS might be suboptimal to capture the relevant structural information for predicting the drug combination sensitivity. However, as the focus of this study was to show the validity of using machine learning methods to predict the CSS score, we decided to explore other chemical fingerprint features as a future step.

**Table 2:** The prediction performances for drug-target features and chemical fingerprint features using Elastic Net. RMSE: root mean square error, R2: coefficient of determination, COR: Pearson correlation, MAE: mean absolute error.

*Feature*	*RMSE*	*R2*	*COR*	*MAE*
*Validated targets*	5.66±1.27	0.77±0.07	0.88±0.04	4.26±0.95
*Validated + predicted targets*	5.70±1.22	0.76±0.06	0.87±0.04	4.34±0.95
*Fingerprints*	6.27±1.19	0.71±0.06	0.85±0.04	4.87±0.94
*Validated targets + fingerprints*	5.30±1.14	0.79±0.06	0.89±0.03	4.07±0.88
*All features*	5.20±1.11	0.80±0.06	0.90±0.03	4.01±0.86

All the values are mean+/-standard deviation.

**Fig 3:**
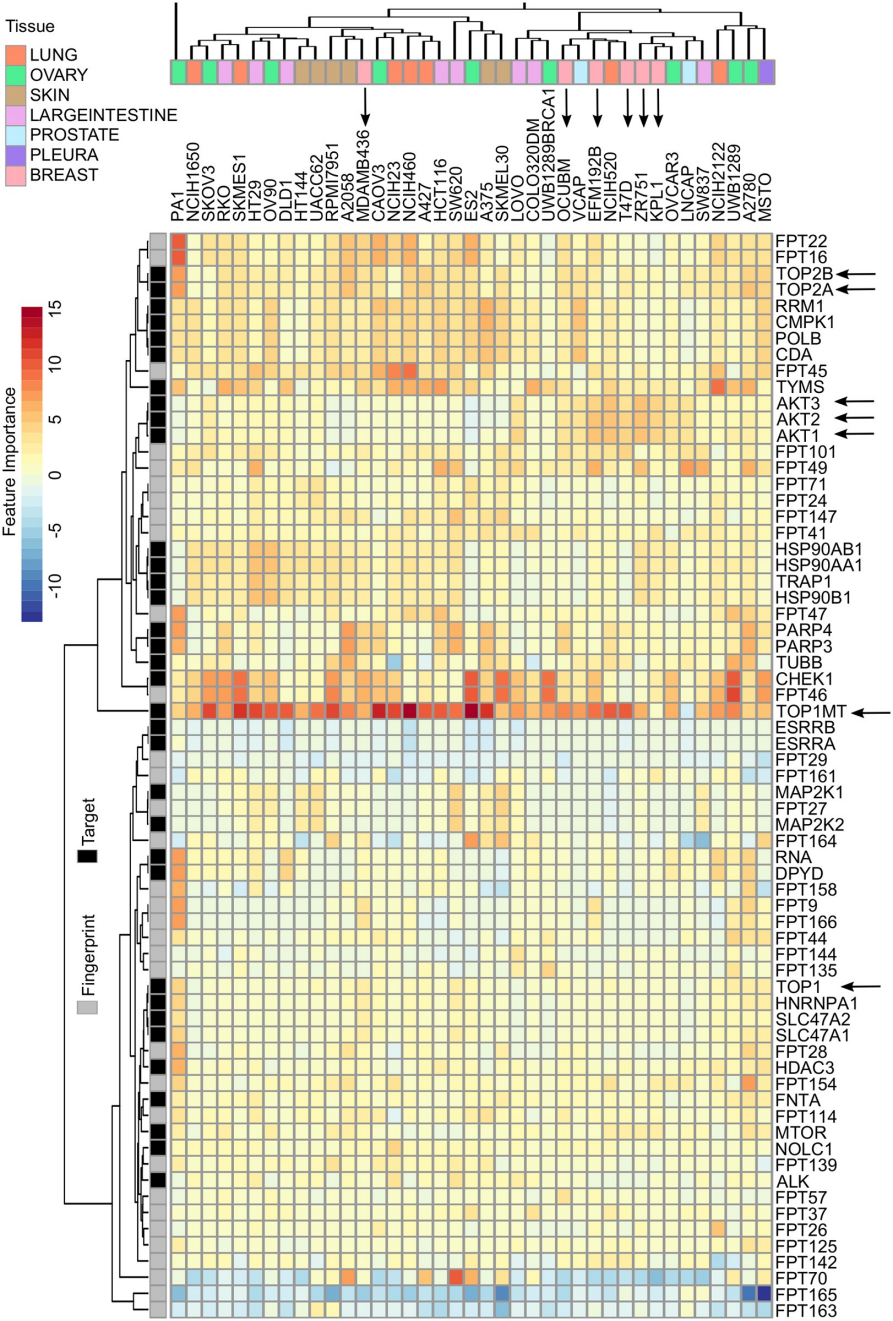
The top important features by Elastic Net for each cell line. Cell-line independent as well as cancer subtype-specific features can be identified by evaluating the regression coefficients of the Elastic Net model. Features such as TOP1MT, TOP2A/B has shown consistently positive coefficients as compared to features such as AKT1/2/3 which showed cancer subtype specificity in breast cancer (indicated as arrows).

We considered the regression coefficients that were determined in the Elastic Net model as an indication of their importance to contribute to the CSS prediction. We found that certain drug target features were present with high coefficients across all the cell lines (Fig 3). For example, DNA topoisomerases including TOP1MT, TOP2A and TOP2B and TOP1 were selected, with average coefficients of 8.2, 2.7, 2.6 and 1.0, respectively. Despite the difference in the level of variable importance, all the DNA topoisomerases showed positive coefficients in 38 of 39 cell lines, suggesting that targeting DNA topoisomerases were associated with a higher CSS. DNA topoisomerases are known proteins which are essential for cell replication and metabolism [37]. Including a topoisomerase inhibitor can thus enhance the drug combination sensitivity in many cancer cell lines. On the other hand, the only cell line that showed negative coefficients for TOP1MT was LNCAP (prostate cancer), which turned out to be the cell line that has the smallest average CSS scores for drug combinations involving the TOP1MT inhibitor (topotecan) (S7 Fig).

If the CSS profiles for two cell lines are similar, then their feature importance vectors are expected to be similar. We focused on the most important features that have their absolute coefficients greater than 3 for at least one cell line, resulting in 67 top features. We then utilized these feature importance scores to cluster the cancer cell lines, using unsupervised hierarchical clustering with the Euclidean metric. We found that cell lines of the same tissue type did not necessarily cluster together, indicating their distinctive drug combination response profiles. For example, we found that breast cancer cell lines did not form a single cluster due to the outlier MDAMB436. Indeed, MDAMB436 is the only triple negative breast cancer (TNBC) subtype, while the other cell lines are either ER positive (KPL1, ZR751 and T47D), or HER2 positive (EFM192B and OCUBM). It has been known that TNBC respond to anticancer drugs differently from ER and HER2 positive breast cancers due to distinct disease mechanisms [38]. The top drug combination features separated these two distinctive breast cancer subtypes, suggesting the validity of using CSS-predictive features to cluster cancers of different subtypes. Furthermore, we found that AKT targets (AKT1/2/3) were among the top features that showed higher importance in the non-TNBC group. A combination of an AKT inhibitor and a TOP1MT inhibitor therefore can be proposed to treat non-TNBC, but not necessarily for TNBC breast cancers. On the other hand, we found that CHEK1 and PARP3/4 targets were selected for the TNBC cell line MDAMB436 but not for the non-TNBC group, suggesting that a combination of a CHEK inhibitor and PARP inhibitor might be effective for TNBC. The mechanisms of actions for the proposed drug combination may prove interesting for experimental validations. Taken together, the pharmaco-features that were determined from the CSS prediction may help pinpoint the underlying target combinations, which are of pivotal importance to explain the drug combination responses.

### The S synergy scores can predict the true synergy and antagonism

Next, we defined the degree of drug synergy as the differences between the dose-response curves of a drug combination and its single drugs. We derived three variants of the S synergy score (*S*_*sum*_, *S*_*max*_, *S*_*mean*_) and compared them with the HSA, Bliss, Loewe and ZIP synergy scores that were determined using the full-dose response matrix. Although being determined using only one row and one column from the dose-response matrix, all the S synergy scores managed to obtain a good correlation with the synergy scores based on the full matrix design (Table 3).

**Table 3:** Pearson correlations of the S synergy scores with those derived using four reference models that were calculated using the full dose-response matrices.

*Synergy Scores*	*HSA*	*Bliss*	*Loewe*	*ZIP*
*S_sum_*	0.72	0.72	0.46	0.55
*S_max_*	0.71	0.65	0.51	0.49
*S_mean_*	0.65	0.63	0.41	0.44

We found that the S synergy scores correlated relatively well with the HSA and Bliss scores, while the correlation started to decrease when comparing to the Loewe and ZIP scores. Since all the synergy scoring models utilized different assumptions for the reference of no synergy, we therefore did not expect a perfect correlation in such pairwise comparisons. For example, the Bliss model assumes that two non-interactive drugs act independently while the Loewe model assumes two non-interactive drugs act as one drug. Their differences in mathematical models have been discussed in our previous publications, such as [16] and [35].

Of all the three variations of S synergy score, we found that *S*_*sum*_ showed the best correlation with those determined using the full dose-response matrix. As *S*_*sum*_ considers the additive effect of single drug sensitivities as the expectation of no synergy, it thus can be considered a more conservative scoring method compared to *S*_*max*_ and *S*_*mean*_, where the maximal and average effect of single drugs were considered as reference separately. To control the false discovery rate of detecting synergistic combinations, we therefore proposed *S*_*sum*_ as a more appropriate scoring method for the cross drug combination design.

Next, we evaluated the predicting accuracy of the S synergy scores for true synergistic and antagonistic drug combinations. To be able to formulate a binary classification problem, we first selected the true positive and true negative drug pairs by applying stringent criteria to determine the ground truth from the full dose response matrix data. The rationale of the ground truth was based on the assumption that full dose-response matrix can capture the truly synergistic and truly antagonistic drug combinations, as it allows the full factorial testing of all the possible doses for a given drug combination. However, as there exist different models for synergy scoring, we decided to apply the most stringent criteria to determine the ground truth, such that a truly synergistic or truly antagonistic drug combination has to fulfill the criteria of all the four existing synergy scoring models (HSA, Bliss, Loewe and ZIP). Namely, the drug combinations with all the four synergy scores (HSA, Bliss, Loewe and ZIP) higher than 5, or lower than -5, were classified as true synergistic or antagonistic drug combinations, respectively. The threshold of [–5, 5] was determined by the empirical distribution of the synergy scores in the O’Neil data, assuming that most of the drug combinations are non-interactive (S8 Fig). Furthermore, we considered that a synergy score of [–5, 5] range corresponds to a [–5, 5] % inhibition which might be due to experimental variation, as indicated by the analysis of replicates from O’Neil dose-response matrix data (mean standard deviation of % inhibition is 6.6%). Therefore, any synergy score between [–5, 5] may be simply experimental noises. From the O’Neil data, we showed that 78.1% of the drug combinations were within the [–5, 5] range by at least one synergy scoring, suggesting that the majority of the drug combinations indeed should not be reliably considered as true synergistic nor true antagonistic (S8 Fig). As a result, we identified 3,716 true synergistic and 218 true antagonistic drug combinations. All the other drug pairs were considered as non-interactive and thus excluded from the analysis.

**Fig 4:**
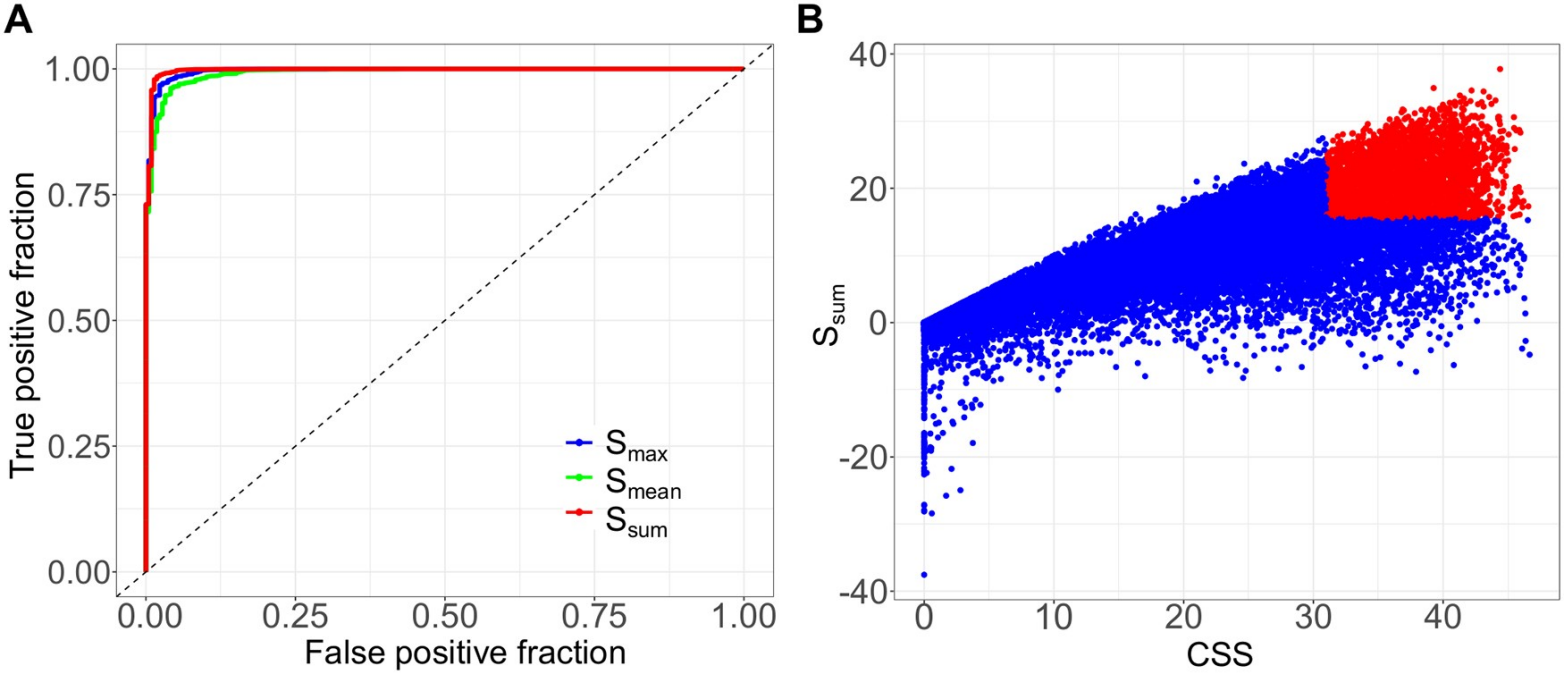
Identification of true synergistic and true antagonistic drug combinations. (A) The ROC curves for the S synergy scores to detect true synergistic and antagonistic drug combinations. (B) The S-S plot for all the drug combinations. The drug combinations with the 75^th^ percentile and above for both the CSS and the S scores were highlighted in red to be considered as the prioritized hits for further experimental validation in a confirmatory screen using a full dose-response matrix design.

Once the ground truth had been determined using the full dose-response matrix data, we then asked the question: Can the S synergy scores that were determined using the cross design correctly identify the most significant synergistic and antagonistic drug combination hits that were confirmed using the full matrix design? We showed that the S synergy scores achieved the area under the ROC curves of 0.997 (*S*_*sum*_), 0.996 (*S*_*max*_) and 0.992 (*S*_*mean*_) to detect the true synergistic and antagonistic combinations (Fig 4A). The area under the precision-recall curves were 0.9997 (*S*_*sum*_), 0.9995 (*S*_*mean*_) and 0.9998 (*S*_*sum*_), suggesting that the S scores retrieved a majority of synergistic combinations with minimal false positive and false negative rates (S9 Fig). The S synergy score was derived from the cross design, where only two vectors of the drug combination responses are needed. Still, the S synergy scores can predict the most synergistic and antagonistic drug combinations with high accuracy. These results showed that the cross design can be reliably utilized as a cost-effective strategy in a primary screen to detect the most significant synergistic or antagonistic drug combinations. On the other hand, the S synergy score and the CSS drug combination sensitivity score utilize the same unit as the percentage of inhibition of cell viability. Therefore, the synergy score can be interpreted as the extra percentage inhibition effect beyond the expectation. We summarized both CSS and S scores for all the drug combinations as an S (sensitivity)-S (synergy) plot (Fig 4B; S4 Table). By applying a threshold of the 3^rd^ quantiles for CSS and S, we can clearly identify the most promising drug combinations that fulfill both the sensitivity and synergy criteria, while avoiding the false positive drug combinations that might be synergistic but do not achieve a sufficient high level of sensitivity (S10 Fig). Taken together, the combined use of CSS drug combination sensitivity score and the S synergy score allows a simultaneous evaluation of the sensitivity and synergy for a drug combination, which will facilitate a more systematic analysis of high-throughput drug combination data with much less experimental materials.

## Discussion

Drug combinations may potentially lead to more durable clinical responses by overcoming intra-tumoral heterogeneity and drug resistance to monotherapies. Identifying drug combinations that are tailored for personalized medicine is a challenge, as the number of possible combinations may easily grow exponentially [39]. High-throughput drug combination screening has been increasingly utilized for early detection of true synergistic and effective drug combinations. However, systematic identification of drug combinations is difficult, as the concepts of synergistic versus effective drug combinations are often intertwined and sometimes interchanged without sufficient clarification. Furthermore, there is a lack of consensus on what the definition of synergy and sensitivity are, which might contribute to the poor reproducibility of many drug combination studies. The uncertainty about the endpoint measurement in drug combination screens brings additional complexity for any machine learning approach to tackle the prediction problem.

We developed a novel scoring approach called CSS for drug combinations that can be efficiently determined using the cross design. We found that the CSS is highly reproducible and therefore can be considered as a robust metric to characterize drug combination sensitivity. To understand the drug combination sensitivity, we implemented a systematic evaluation of the prediction accuracy of three machine learning methods. We showed that machine learning in general worked well for the prediction of CSS, where Elastic Net showed the best performance according to our cross-validation setting. We found that the drug target information for the compounds as well as their chemical fingerprints are highly predictive of the CSS values, with an accuracy comparable to the level of experimental replicates. Therefore, the rationale of considering a drug combination as a function of their target and fingerprint profiles can be justified. This would also allow the augmentation of single-drug screening and drug combination screening data together to train a machine learning model, as many drugs are multi-targeted which are equivalent to a drug combination with the same target profile. In our study, we utilized the SEA method to predict new targets of compounds, by applying a combination of thresholds of Z-score, Tanimoto coefficient and p-value suggested by the authors of SEA [25]. In addition to the predicted targets, we also included the known primary and secondary targets of the compounds, so that the risk of false negative is minimal. However, we could not find significant improvement on the prediction accuracy when including the SEA-predicted secondary targets (Table 2), suggesting that the unknown targets for a compound may contribute minimally to the drug combination sensitivity. In this study we focused on drug combination prediction within the same cell line. In the future, we could include the molecular features of the cell lines to improve the prediction accuracy, aiming to identify drug combination specific biomarkers across different cell lines. On the other hand, as the focus of the study is to propose the new experimental design and to justify its associated drug combination scoring methods, we tested the predictability of CSS using only conventional machine learning methods with standard cross-validation schemes. A more comprehensive evaluation of machine learning approaches may be developed by including multiple cross-validation schemes and data pre-filtering techniques. More advanced machine learning methods such as Deep Learning [13] or network-based methods [40] may further improve the prediction accuracy as well as help the biological understanding of the mechanisms of action.

A truly promising drug combinations shall reach sufficient therapeutic efficacy via a strong sensitivity and synergy. Therefore, both these aspects should be evaluated for the prioritization of most potential drug combination hits. While there have been multiple synergy scoring methods that can be applied to the full matrix design, they do not always produce consistent results. The truly synergistic and antagonistic drug combinations may therefore be determined by achieving the consensus across the different scoring methods [16]. Based on the CSS drug combination sensitivity scoring, we developed an S synergy score to quantify the degree of interactions in a drug pair, and showed that it can identify truly synergistic and antagonistic drug combinations accurately. Tailored for the cross design, the S synergy score can be used for the prioritization of a primary drug combination screen, after which only the most significant drug combinations should warrant a confirmation screen using the full matrix design. Furthermore, we proposed a novel S-S plot (Fig 4B) to visualize drug combination sensitivity and synergy using the same scale, which enables an unbiased way to explore high-throughput drug combination data more efficiently with minimal bias. Notably, the CSS is defined at the IC_50_ concentrations of the background drugs. Therefore, a synergistic drug combination determined by the S score should be more therapeutically relevant than a drug combination where the synergy is detected at higher concentrations, which are often associated with unwanted off-target effects and side-effects.

The proposed cross design aimed for alleviating the limitation of the conventional dose-response full matrix design which usually requires a large amount of cancer cells that may not be amenable especially from patient-derived samples. Empowering the cross design with the CSS and S scoring methods, we are foreseeing a lower technical barrier to carry out large-scale drug combination studies with minimal cellular materials. Although we showed the proof-of-concept using the drug combination data involving cancer cell lines, the cross design coupled with the CSS and S scoring methods can be readily applied for ex_vivo drug screening, where the amount of patient-derived materials can be extremely limited and technically difficult to obtain due to culture constraints [41]. While the majority of drug combination screens are limited to cytotoxic and molecularly targeted molecules, the cross design should be also favored for the testing of combinations that involve immunotherapies and antibodies. Furthermore, the cross design is not only applicable for cancer but also for other diseases, as long as cellular phenotypes of interests can be measured at multiple dose levels. With the help of cross design and its data analysis tools, drug combination discovery can be more quickly advanced and may eventually lead to the validation of personalized drug combinations in clinical trials.

## Supporting information

S1 Table Drug target profiles including experimentally-validated primary and secondary targets, and SEA-predicted secondary targets for the 38 compounds. (XLSX)

S2 Table The chemical information including MACCS fingerprint profiles for the 38 compounds. (XLSX)

S3 Table The Pearson correlations of the CSS values obtained using the average of four viability replicates, with the CSS values obtained from the replicate separately. (XLSX)

S4 Table CSS drug combination sensitivity scores and S synergy scores for each drug combination. (XLSX)

S1 Fig The relation between original *AUC* and normalized *AUC*′. *AUC* is defined as the area under the log10 transformed drug combination dose response curve on the foreground drug concentration interval [*c*_1_, *c*_2_], while *AUC*′ normalizes *AUC* by subtracting the area of the rectangular box with the height of minimal inhibition (%min) considered to be experimental noise, and then scaled by the factor of (1 − %min)(*c*_2_ − *c*_1_).
(TIF)

S2 Fig The heatmap of the CSS_1_-CSS_2_ correlations sorted by drug combinations. Drug classes are determined by their primary targets.
(TIF)

S3 Fig The scatter plot of the CSS_1_ and CSS_2_ values obtained using permuted data. The color of the data points represents the tissue of origin.
(TIF)

S4 Fig Reproducibility of CSS values over the replicates. The line plot of the minimal and maximal values for the CSS replicates combined with CSS values over the standard deviation of the CSS replicates.
(TIF)

S5 Fig The coefficient of variation (CV) for each drug in the single drug screens. The Pearson correlations between CSS_1_ and CSS_2_ for the drug combinations that involve a given drug were shown on top of each bar.
(TIF)

S6 Fig The Pearson correlation between CSS_1_ and CSS_2_ after filtering out the drug combinations that have absolute difference between CSS_1_ and CSS_2_ over than a given threshold. The threshold of 10 achieved a correlation (0.93) close to the midpoint (0.91) of the range.
(TIF)

S7 Fig The Pearson correlation between the variable importance of TOP1MT and the average CSS for TOP1MT inhibitor for all the 39 cell lines. LNCAP is the only line which has a negative variable importance for TOP1MT.
(TIF)

S8 Fig The distribution of (A) minimal and (B) maximal of Loewe, Bliss, HSA and ZIP synergy scores derived from the dose-response matrix data. True synergistic combinations were determined as the minimal of the four scores higher than 5 while true negative combinations have the maximal of the four scores lower than 5, resulting in 20.7% and 1.2% of the total drug combinations respectively.
(TIF)

S9 Fig The precision-recall curves for the S synergy scores to predict true synergistic (positive) and true antagonistic (negative) drug combinations, demonstrating that the high true positive rate is not a consequence of imbalanced classes. (TIF)

S10 Fig Examples of the synergistic but not sensitive drug combinations. All the drug combinations shown here have an S synergy score higher than 5, while their CSS score lower than 10. The cell lines are colored based on their tissues of origin. Bar plot in the inset shows the mean % inhibition that can be achieved by these drug combinations (denoted as false positive group), as compared to the top 100 drug combinations ranked by CSS (denoted as true positive group).
(TIF)
