# Supplementary figures and images for: Drug combination sensitivity scoring facilitates the discovery of synergistic and efficacious drug combinations in cancer
Source: PLoS Comput Biol. 2019 May 20;15(5):e1006752. doi: 10.1371/journal.pcbi.1006752 (PMC6544320; doi:10.1371/journal.pcbi.1006752)

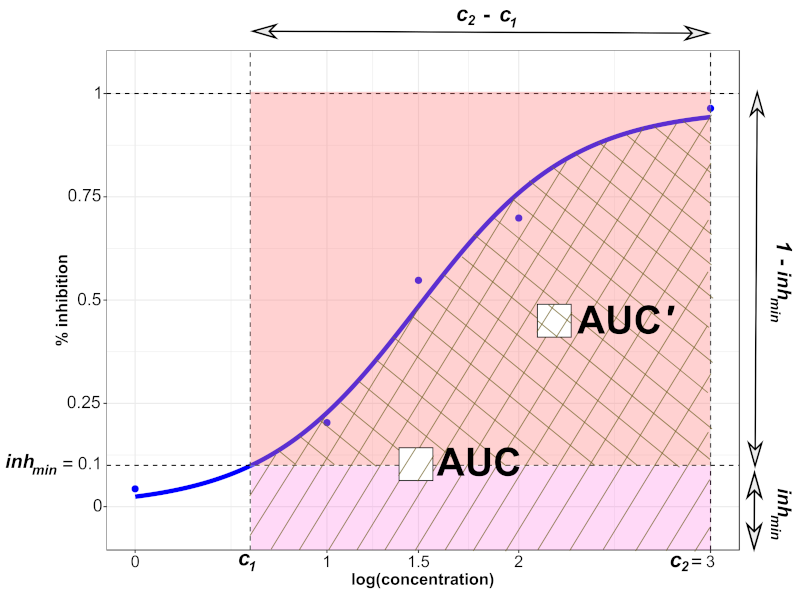

Supplement: S1 Fig — AUC is defined as the area under the log10 transformed drug combination dose response curve on the foreground drug concentration interval [c1, c2], while AUC′ normalizes AUC by subtracting the area of the rectangular box with the height of minimal inhibition (%min) considered to be experimental noise, and then scaled by the factor of (1 − %min)(c2 − c1). (TIF) [file pcbi.1006752.s005.tif]

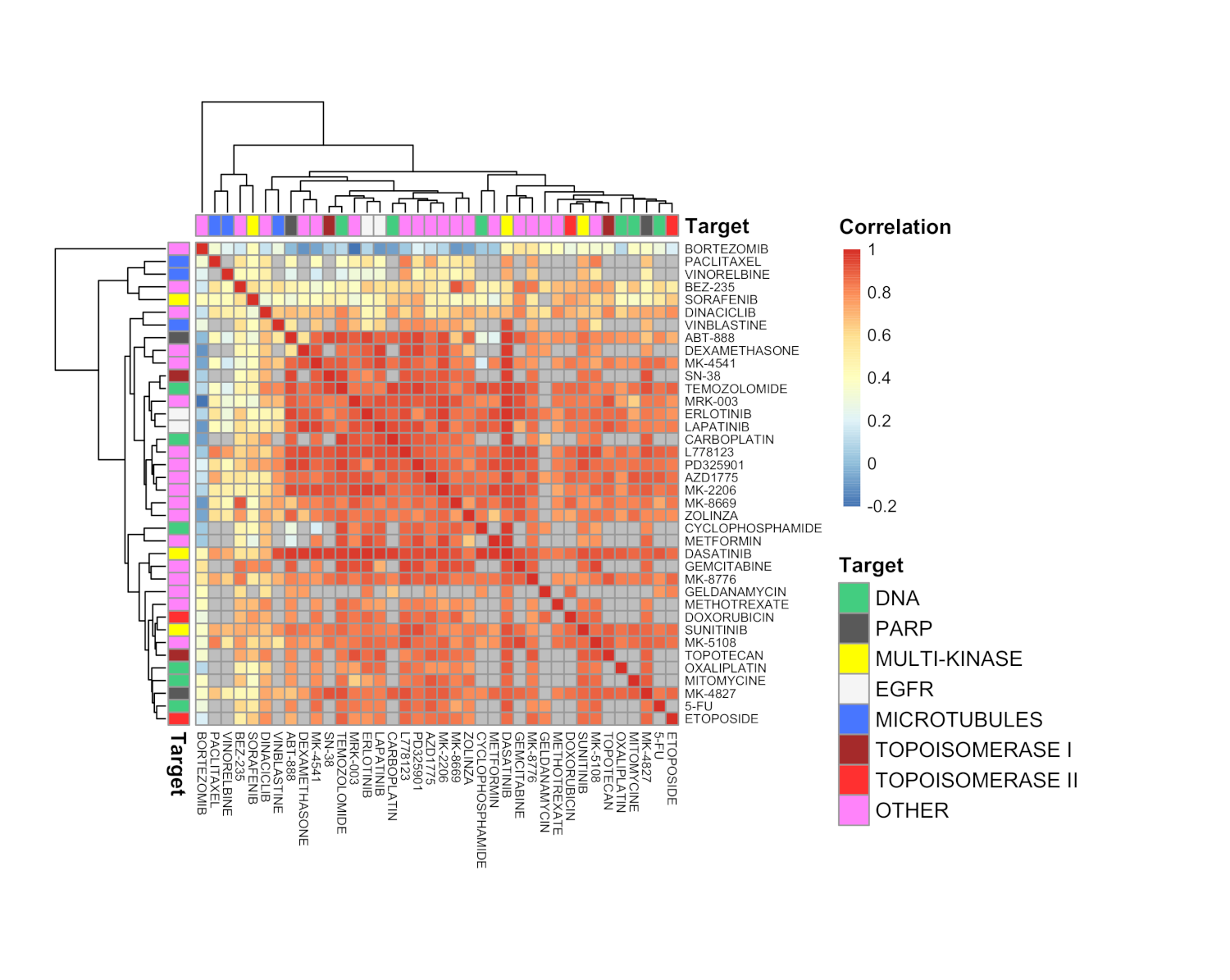

Supplement: S2 Fig — Drug classes are determined by their primary targets. (TIF) [file pcbi.1006752.s006.tif]

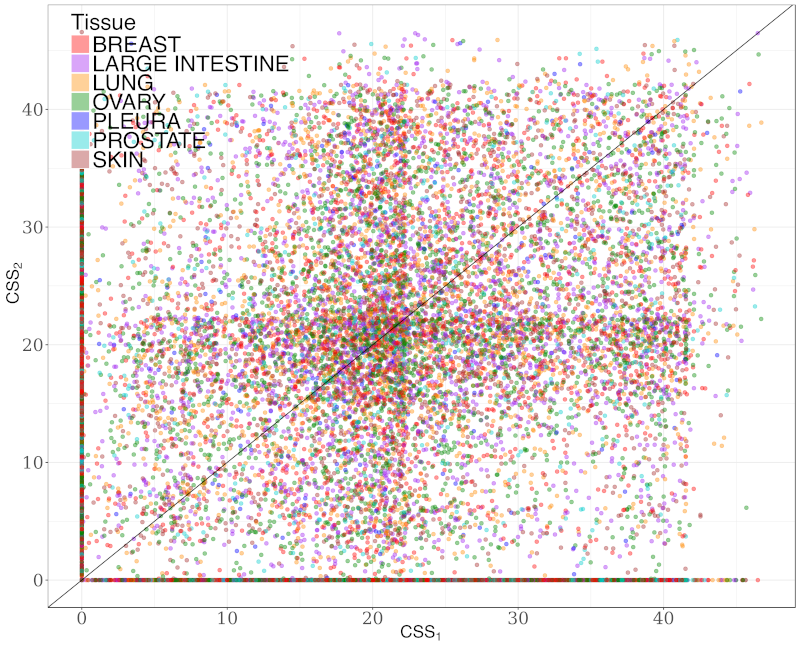

Supplement: S3 Fig — The color of the data points represents the tissue of origin. (TIF) [file pcbi.1006752.s007.tif]

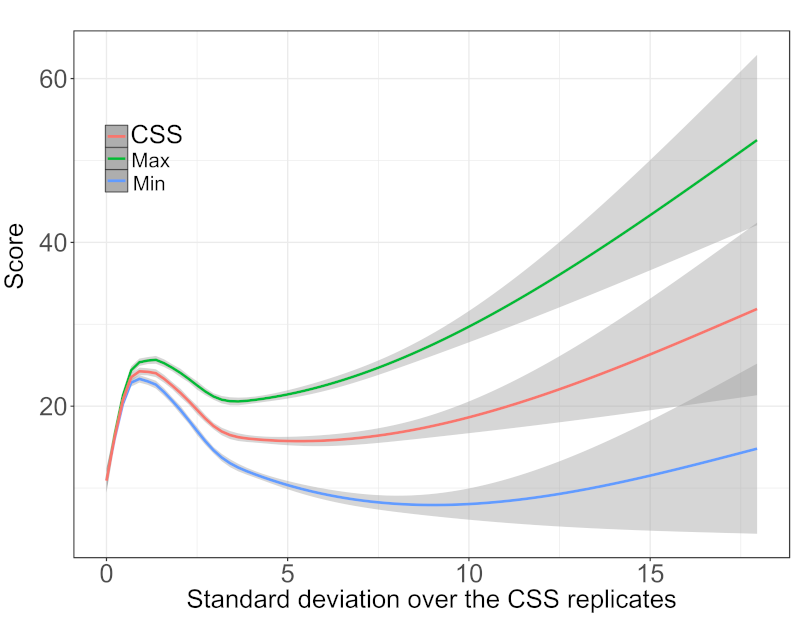

Supplement: S4 Fig — The line plot of the minimal and maximal values for the CSS replicates combined with CSS values over the standard deviation of the CSS replicates. (TIF) [file pcbi.1006752.s008.tif]

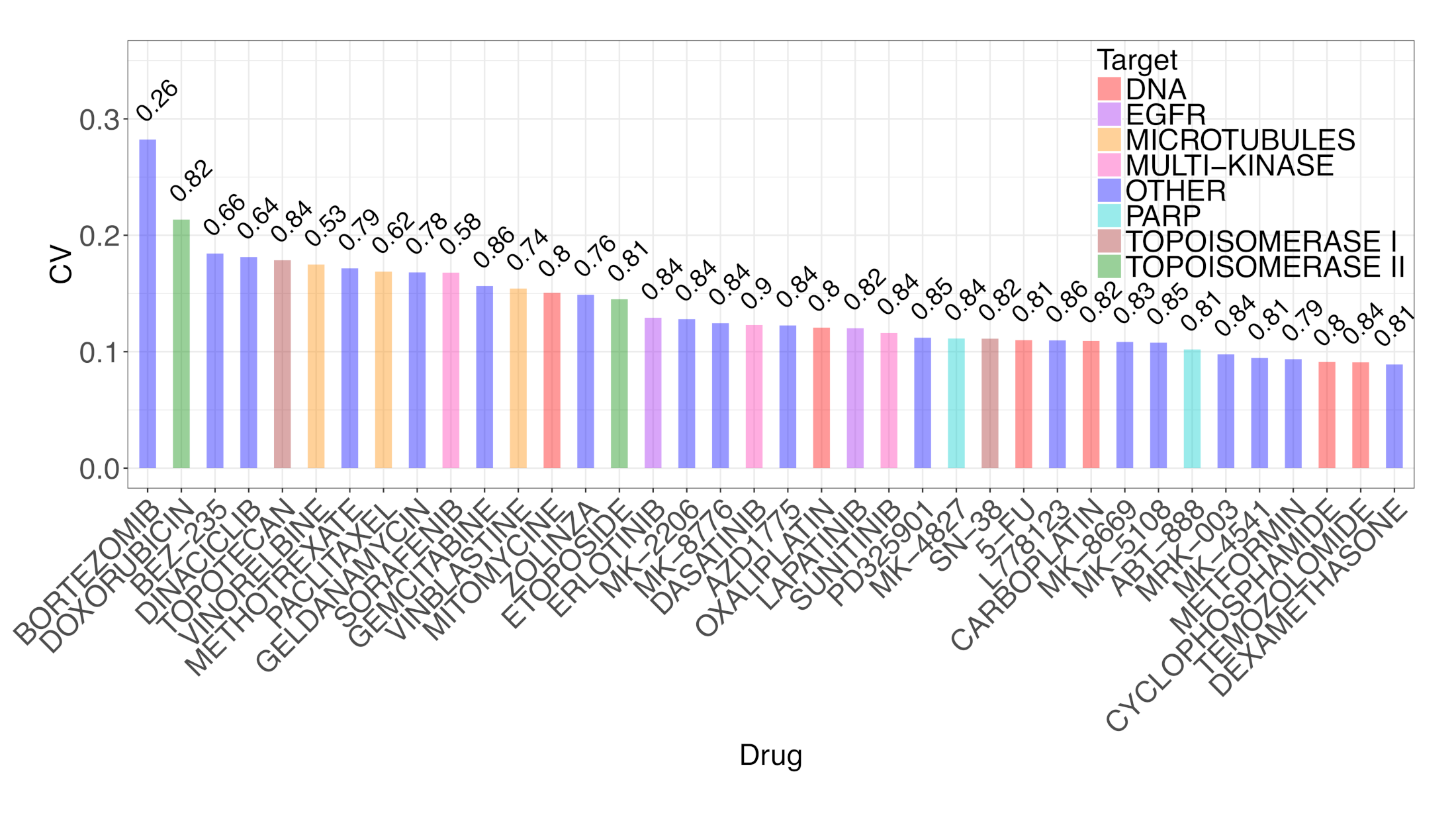

Supplement: S5 Fig — The Pearson correlations between CSS1 and CSS2 for the drug combinations that involve a given drug were shown on top of each bar. (TIF) [file pcbi.1006752.s009.tif]

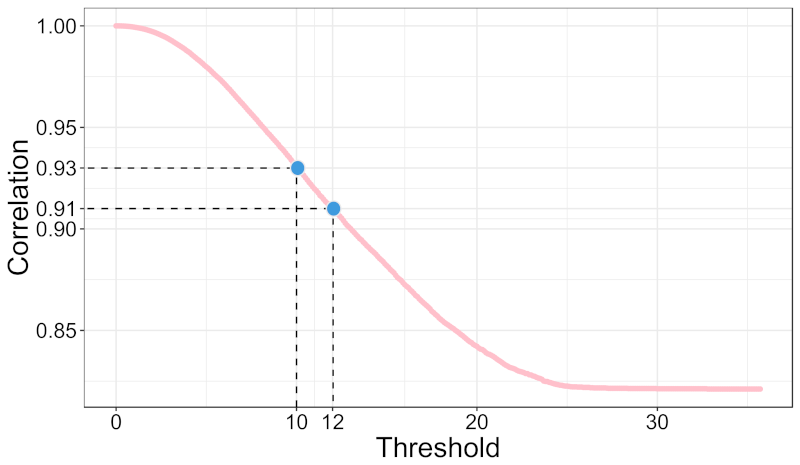

Supplement: S6 Fig — The threshold of 10 achieved a correlation (0.93) close to the midpoint (0.91) of the range. (TIF) [file pcbi.1006752.s010.tif]

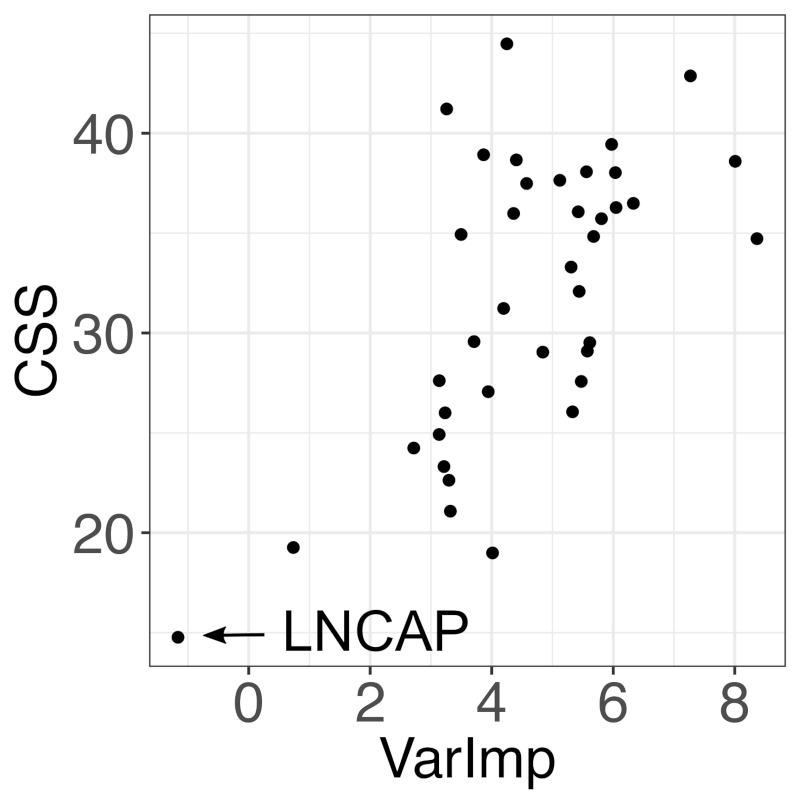

Supplement: S7 Fig — LNCAP is the only line which has a negative variable importance for TOP1MT. (TIF) [file pcbi.1006752.s011.tif]

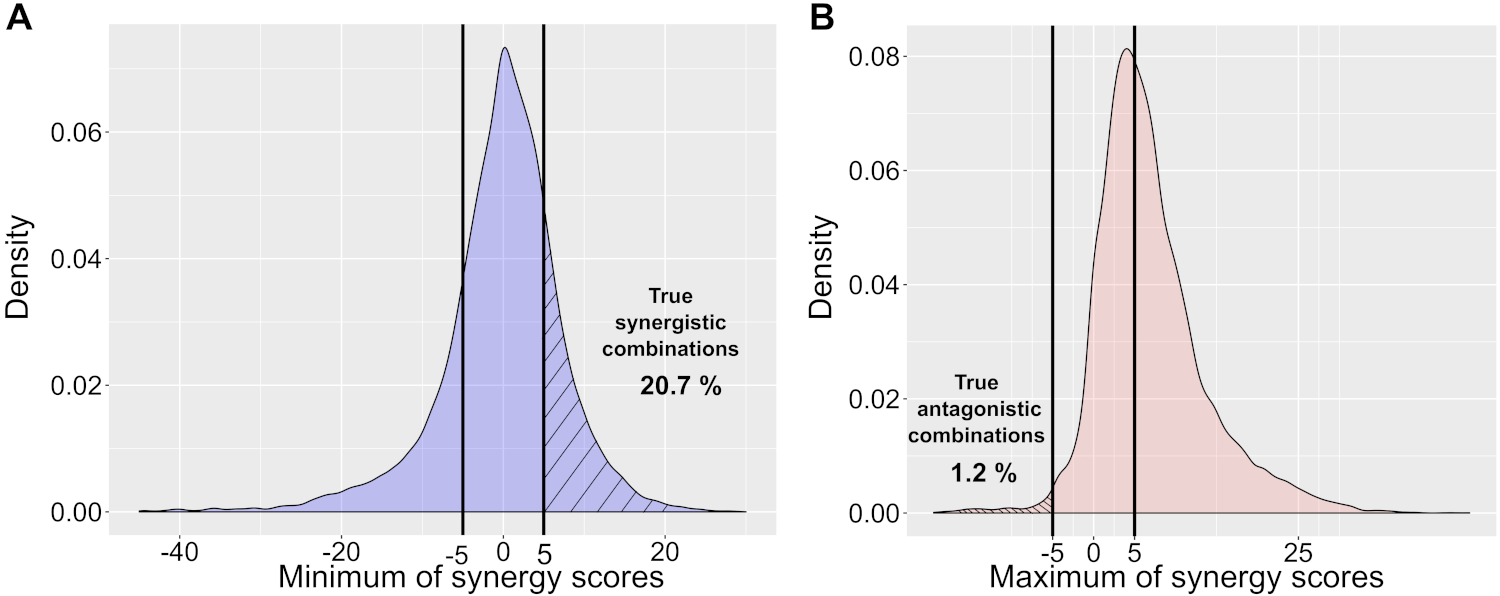

Supplement: S8 Fig — True synergistic combinations were determined as the minimal of the four scores higher than 5 while true negative combinations have the maximal of the four scores lower than 5, resulting in 20.7% and 1.2% of the total drug combinations respectively. (TIF) [file pcbi.1006752.s012.tif]

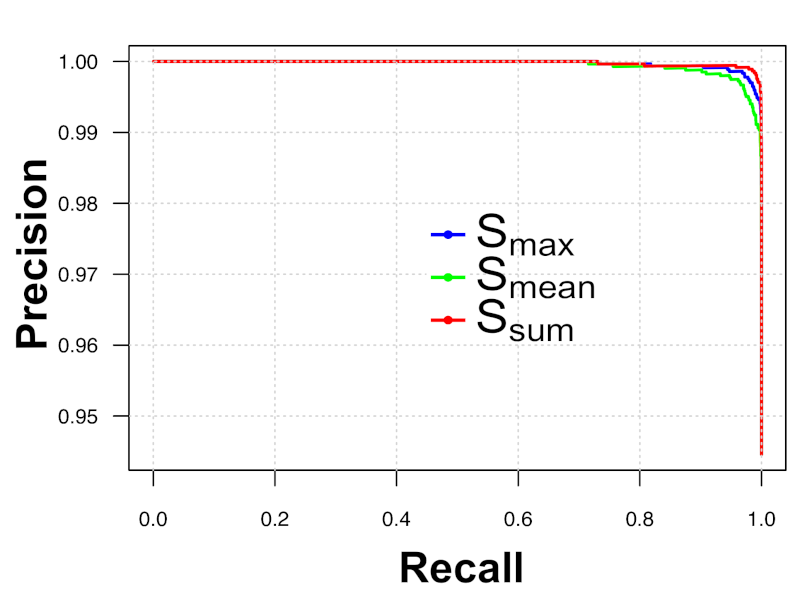

Supplement: S9 Fig — (TIF) [file pcbi.1006752.s013.tif]

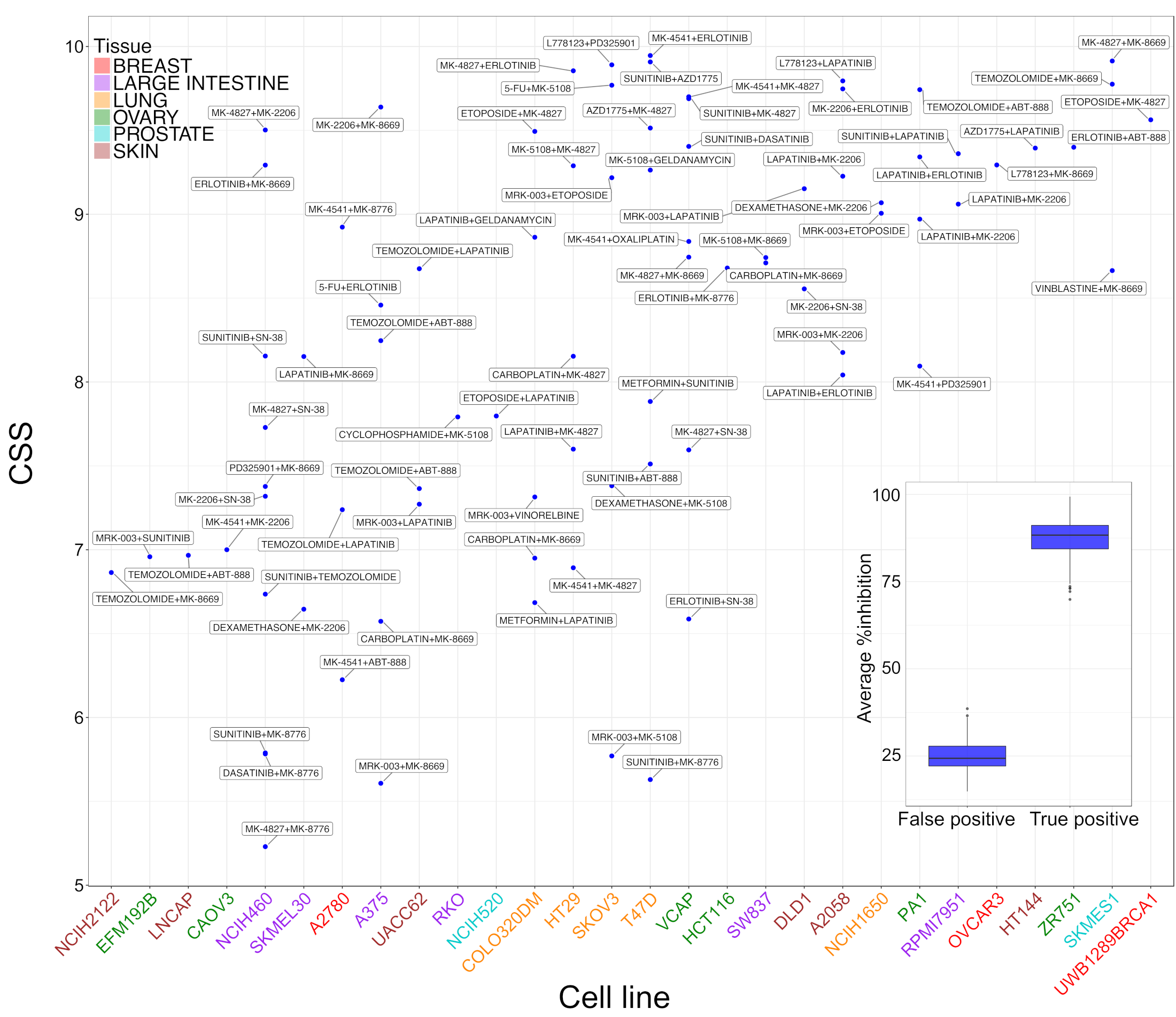

Supplement: S10 Fig — All the drug combinations shown here have an S synergy score higher than 5, while their CSS score lower than 10. The cell lines are colored based on their tissues of origin. Bar plot in the inset shows the mean % inhibition that can be achieved by these drug combinations (denoted as false positive group), as compared to the top 100 drug combinations ranked by CSS (denoted as true positive group). (TIF) [file pcbi.1006752.s014.tif]
